# The ins and outs of sporozoite biology in the dermis

**DOI:** 10.1186/1475-2875-13-S1-O6

**Published:** 2014-09-22

**Authors:** Christine Hopp, Photini Sinnis

**Affiliations:** 1 Johns Hopkins Bloomberg School of Public Health, USA

## 

*Plasmodium* sporozoites are inoculated by infected mosquitoes into the dermis of the mammalian host. Once in the skin, sporozoites use gliding motility to move within the dermis and find a blood vessel. They then breach the endothelial barrier, enter the bloodstream and go to the liver. Several mutants that we have generated as well as recent antibody-inhibition studies suggest that the skin is a bottleneck for the parasite and possibly one of our best opportunities to intervene. Using the rodent malaria model, we have begun to analyze sporozoite movement in the skin and the innate immune response to sporozoites delivered by mosquito bite. These data and their relevance to the malaria vaccine effort will be discussed.

